# First record of the invasive Asian fish tapeworm *Bothriocephalus acheilognathi* in Honduras, Central America

**DOI:** 10.1051/parasite/2015007

**Published:** 2015-02-06

**Authors:** Guillermo Salgado-Maldonado, Wilfredo A. Matamoros, Brian R. Kreiser, Juan Manuel Caspeta-Mandujano, Edgar F. Mendoza-Franco

**Affiliations:** 1 Universidad Nacional Autónoma de México, Instituto de Biología, Laboratorio de Helmintología, Ciudad Universitaria México, D. F.; 2 Department of Biological Sciences, University of Southern Mississippi Hattiesburg MS 39406 USA; 3 Universidad Autónoma del Estado de Morelos, Facultad de Ciencias Biológicas y Centro de Investigaciones Biológicas, Laboratorio de Parasitología de Animales Silvestres, Avenida Universidad 1001, Colonia Chamilpa, Cuernavaca Morelos México; 4 Universidad Autónoma de Campeche, Instituto de Ecología, Pesquerías y Oceanografía del Golfo de México (EPOMEX), San Francisco de Campeche Campeche México

**Keywords:** Río Nacaome, Central America, Invasive species, Parasites, ITS-1, ITS-2

## Abstract

This paper provides the first report of the invasive Asian fish tapeworm, *Bothriocephalus acheilognathi* Yamaguti, 1934, in Honduras. The cestode was found in *Profundulus portillorum* (Cyprinodontiformes: Profundulidae), which represents a new host record, and which is a member of a genus faced with a variety of conservation challenges, now potentially complicated by the presence of this pathogenic cestode. Nearly complete sequence data from the ITS-1 5.8S and ITS-2 regions corroborate the determination based on morphological characteristics. Several species of carp were introduced to Honduras for aquaculture purposes in the early 1980s and the presence of the Asian fish tapeworm in Honduras may be related to these introductions. In addition, this report documents the currently known geographical distribution of this parasite in Central America, first recorded from Panamá and now from Honduras.

## Abstract (fr)

Cet article présente la première mention du cestode de poissons invasif asiatique *Bothriocephalus acheilognathi* Yamaguti, 1934 au Honduras. Le cestode a été trouvé chez *Profundulus portillorum* (Cyprinodontiformes: Profundulidae), un nouvel hôte, qui est membre d’un genre auquel se posent de nombreux problèmes de conservation, désormais potentiellement compliqués par la présence de ce cestode pathogène. Les données des séquences presque complètes des régions ITS-1 5.8S et ITS-2 corroborent la détermination sur la base de caractéristiques morphologiques. Plusieurs espèces de carpes ont été introduites au Honduras à des fins d’aquaculture dans le début des années 1980 et la présence du cestode de poissons asiatique au Honduras pourrait être liée à ces introductions. En outre, cet article rapporte la répartition géographique actuelle connue de ce parasite en Amérique centrale, d’abord signalé au Panama et maintenant au Honduras.

## Introduction

The Asian fish tapeworm *Bothriocephalus acheilognathi* Yamaguti, 1934 has been widely disseminated around the world. Initially, in the 1960s–1970s in the former USSR and eastern European countries, the main vector of its spread appears to be the introduction of its native host, the Asian grass carp *Ctenopharyngodon idella*, for aquacultural purposes or for use in the control of aquatic vegetation. At present, it is probable that common carp, koi carp, mosquito fish, and probably many other fish serve as the main vehicle of expansion of this parasite. This cestode is notorious for exceptionally low specificity to definitive hosts. As an adult, it lives in the gastrointestinal tract of more than 200 freshwater fish species, and has also been reported from amphibians, reptiles, and birds around the world [35, 42].

The native range of *B*. *acheilognathi* probably only includes the Amur River forming the border between China and eastern Russia [8]. However, anthropogenically aided, this species has spread to all continents except Antarctica [35]. Other remarkable records include islands such as the British islands [2], Puerto Rico [6], Mauritius [26], and even as remote as Hawaii [15–17, 40]. In North America, it has only been reported from two regions of Canada [7, 23] and from several regions across the United States [7], but it is broadly distributed in Mexico [29, 31]. However, *B*. *acheilognathi* has only been reported once in South America from the introduced Asian carp *Cyprinus carpio* in a fish farm in northeastern Brazil [13, 27, 35]. Most recently, it has been recorded from Central America, in Panama [9].

This cestode is an important pathogen of wild, feral, and cultured fish [8, 20, 21, 35]. Thus, it is considered of great importance for commercial production and hatchery operations. However, it is also of special concern for conservation and freshwater fish management, due to the fact that its pathogenicity is a threat to populations of native and endemic freshwater fishes [5, 10–12, 19, 21, 31].

In Eurasia and Africa, *B*. *acheilognathi* has mainly remained restricted to cyprinids [8, 28]. However, in North America the parasite has colonized non-cyprinid hosts, with new host records and range extensions still being reported [3, 4, 32, 41]. Based on the extremely wide spectrum of fish hosts, some belonging to different families and even different orders of fishes, accurate identifications of *B*. *acheilognathi* are required to fully establish its propensity for host shifts. Likewise, determining the potential threat of newly introduced parasites to imperiled species depends on accurate identifications. The application of molecular data provides a means to verify identifications [e.g., 4, 9, 22].

The goal of this work was to document the first record of *B*. *acheilognathi* in profundulid freshwater fishes in Honduras, Central America. Profundulidae is the only freshwater fish family that originated in Central America [25, 39]. With only eight known species, mostly confined to high altitude stream habitats between southern Mexico and Honduras, this group includes species of conservation concern that are already experiencing declines from other environmental factors [24, 25].

## Materials and methods

### Study site, fishing gear, and parasite sampling

**Figure 1.**
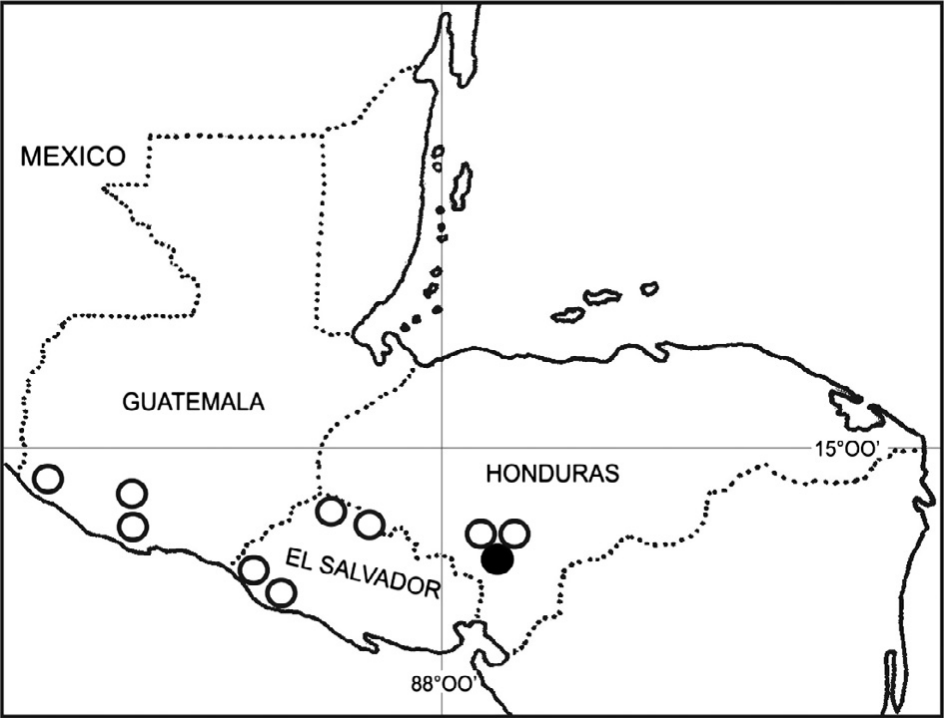
Map of *Profundulus* spp. collections in Central America (circles). The black circle depicts the single locality in which *B. acheilognathi* was collected (Nacaome River) within *Profundulus portillorum*.

We visited 10 localities in Central America (Guatemala, El Salvador, and Honduras; Fig. 1) in May of 2014. Fishes were collected with the use of an electrofishing device, dip-nets, and seines; they were transported live to the laboratory and inspected for helminths within 24 h post-capture. They were examined under a dissecting microscope. Helminths were fixed either in hot 4% formalin for staining and whole mounting, or in 95% ethanol for molecular procedures. Two cestodes were stained with Mayer’s paracarmine, dehydrated using a graded alcohol series, cleared in methyl salicylate, and mounted whole. Voucher specimens were deposited in the “Colección Nacional de Helmintos (CNHE)” at the Instituto de Biología, Universidad Nacional Autónoma de México (CNHE Catalog No. 9368).

### Molecular procedures

Total genomic DNA was extracted from two individuals identified via morphology as *B*. *acheilognathi* with a DNeasy Tissue Kit (QIAGEN Inc., Valencia, CA, USA) and DNA was then stored at −20 °C until use. The ITS-1.5.8S and ITS-2 regions was amplified using the BD1 and BD2-A primers as reported in other studies [9, 22]. Polymerase chain reactions (PCRs) were performed in 25 μL reaction volumes consisting of 1× reaction buffer (New England Biolabs, Beverly, MA, USA), 200 μM dNTPs, 2 mM MgCl_2_, 0.5 units of Taq polymerase (New England Biolabs), 0.3 μM of each primer, approximately 20 ng template DNA, and water to the final volume. Cycling conditions consisted of an initial 1 min denaturing step at 95 °C followed by 30 cycles of 1 min at 95 °C, 1 min at 55 °C, and 1 min at 72 °C with a final elongation step of 7 min at 72 °C. PCR products were cleaned with ExoSAP-IT (USB Co., Cleveland, OH, USA), and sequencing was performed by Eurofins Genomics (Louisville, KY, USA) using the primers described above.

Sequence data were edited using Sequencher v. 4.10.1 (GeneCodes Co., Ann Arbor, MI, USA), and the sequences generated by this study were aligned with those *Bothriocephalus acheilognathi* from Luo et al. (2002; GenBank Accession Numbers AF362408–AF362433) as well as one sequence from *B. claviceps* (AF362434) to serve as the outgroup. The sequence from Choudhury et al. (2013; JN632481) was not included in the alignment as it represented only a portion of the region sequenced. The presence of microsatellite repeats in these sequences made alignment difficult. The initial alignment was performed via Sequencher v. 4.10.1 and was then revised by eye. The best model of molecular evolution for these sequences was selected as the HKY+G by MEGA6 [36]. This model was then used in a maximum likelihood (ML) analysis with the deletion of positions containing gaps with branch support assessed by bootstrapping (1000 replicates; [14]).

## Results

A total of 215 individuals of *Profundulus* spp. (Cyprinodontiformes: Profundulidae) were examined from 10 localities of three Central American countries (Fig. 1): *Profundulus guatemalensis* (Günther) (*n* = 80) from three localities in Guatemala; *P. kreiseri* Matamoros, Schaefer, Hernández, and Chakrabarty (*n* = 95), from four localities in El Salvador; and *P. portillorum* Matamoros and Schaefer (*n* = 40) from three localities in Honduras. Five individual cestodes (*B. acheilognathi*) were found parasitizing 1 of 30 *P. portillorum* in an unnamed creek in the town of Ojojona in the Department of Francisco Morazán (13°55′43.7ʺ N, 87°17′40ʺ W), Río Nacaome drainage basin, Honduras; no exotic fishes were found at this locality. Other examined fishes from several localities of Central America include six *Poecilia sphenops* Valenciennes, four *Poeciliopsis pleurospilus* (Günther) (Poeciliidae); two *Rhamdia laticauda* (Kner) (Heptapteridae), and two *Agonostomus monticola* (Bancroft) (Mugilidae); none of these resulted positive for *B*. *acheilognathi*.

The cestodes were identified as *Bothriocephalus acheilognathi* based on the following combination of characters (see [34]), worms moderately large 11.7–14.5 mm total length (two specimens measured in whole mounts); a scolex heart shaped, 574–594 × 465–663 μm with dorsally and ventrally bothria short and very deep; first proglotids immediately posterior to scolex, neck absent, the posterior maturing proglottids acraspedote, the proglottids having rounded edges, testes medullary, oval to spherical 63–78 in number; ovary lobed, median, near posterior margin of proglottids. Eggs were operculate and unembryonated 45–60 × 33–37 μm (10 eggs measured).

**Figure 2.**
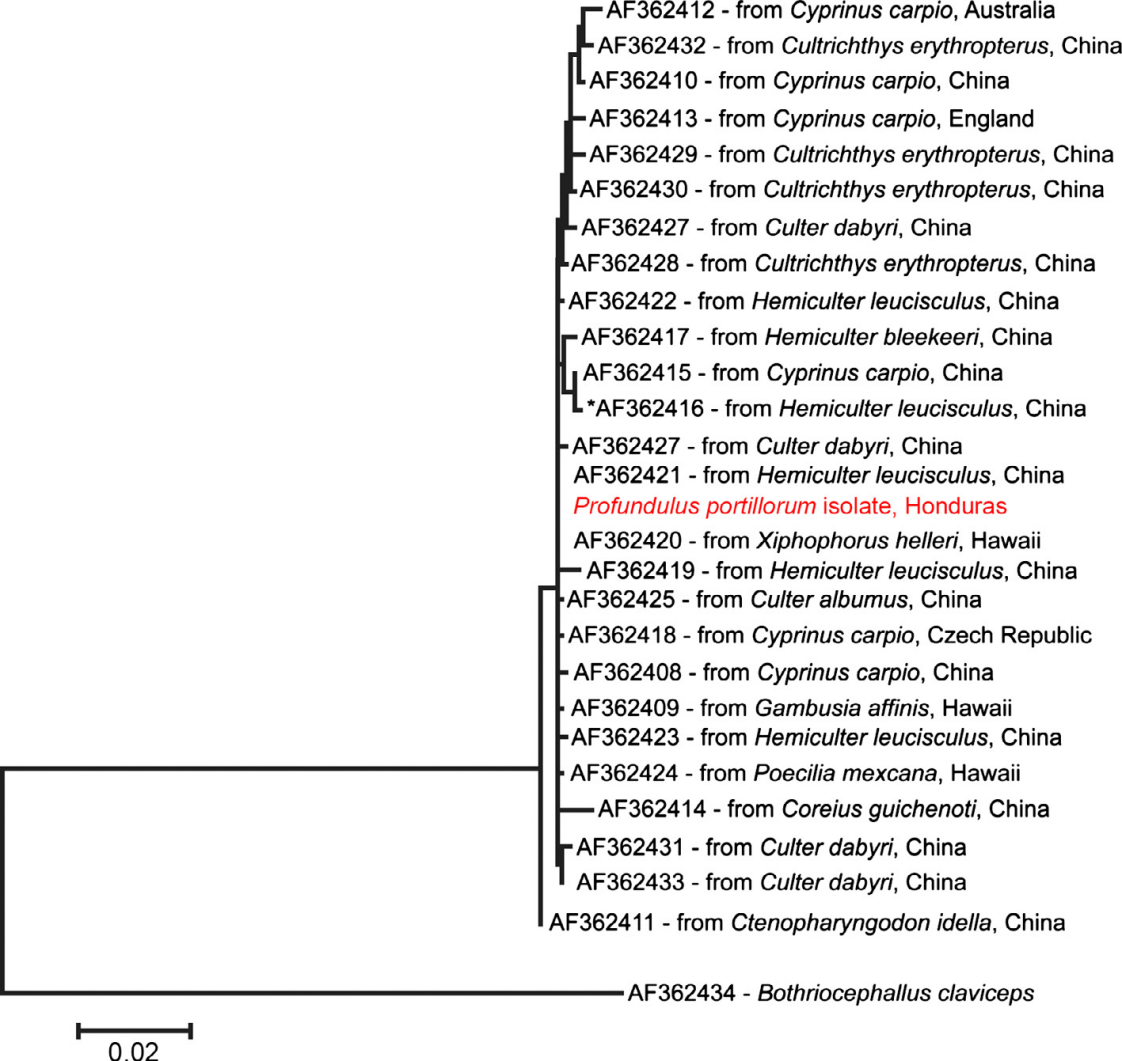
The phylogenetic tree produced by the maximum likelihood analysis of sequences used in this study. Sequences from Luo et al. (2002) are identified by their GenBank Accession Number, the fish species from which the specimen was isolated, and the geographic location of the collection. The label “*Profundulus portillorum* isolate” identifies the sample from Honduras. The asterisk marks the only branch within the ingroup with greater than 85% bootstrap support.

A molecular analysis of two individual *B. acheilognathi* was performed to verify identification. After editing, we generated sequences of 1315 bp, which were identical for the two individuals. This sequence has been submitted to GenBank (KP099579). The completed alignment of 26 sequences from Luo et al. [22] and our sequence comprised of 1424 positions. When gaps were excluded there were a total of 47 variable sites among the *B. acheilognathi* sequences. Our sequence was most similar to AF362420 (isolated from *Xiphophorus helleri* from Kahana Stream, Oahu, Hawaii; Fig. 2) and AF362421 (isolated from *Hemiculter leucisculus* from Honghu Lake, Hubei, China; Fig. 2). These sequences appear to be identical in the ML tree (Fig. 2) since the two bases that differed in our sequence occurred in regions with gaps and were excluded from the ML analysis. Our sequence is also nearly identical to the one from Panama (Choudhury et al. [9]; JN632481) with the exception of the addition of one extra TGAG repeat within our sequence starting at base position 809 in JN632481. The overall topology of the ML tree (Fig. 2) is weakly supported as only one of the internal branches had more than 85% bootstrap support. The lack of strong phylogenetic support does not impact our use of these data since our goal was to verify the identification of our specimen and not to produce a robust phylogeny for the group.

## Discussion

In this work, we report for the first time the presence of the Asian fish tapeworm, *B. acheilognathi*, in Honduras, with the freshwater fish *P*. *portillorum* being a new definitive host of this parasite. In general, the number of global introductions is rising, and freshwater ecosystems are particularly susceptible to invasions because of their connectivity [18]. Given the fact that alien species can potentially spread rapidly through connected river systems, early detection is vital if managers are going to attempt to monitor and/or control the spread of the invader. Thus, the record of *B*. *acheilognathi* from *P*. *portillorum* in freshwater bodies of Honduras at least indicates the capacity of the cestode to invade this region and provides a starting point for future efforts to explore the historical and ecological contexts of this introduction. The present data confirm that the host record and geographical distribution of this cestode is still increasing. Recent surveys [37–39] show that congeneric *P*. *hildebrandi* Miller, a threatened species, are heavily parasitized by *B*. *acheilognathi* in southern Chiapas in Mexico, constituting a concern for its conservation status. However, our surveys of other Mexican *Profundulus* have failed to detect *B*. *acheilognathi* in other species.

It has been widely suggested that the spread of *B. acheilognathi* is related to the introduction of the grass carp, *Ctenopharyngodon idella*, which is *B. acheilognathi*’s principal host in its native distribution. Several species of carp (i.e. *Ctenopharyngodon idella, Hypophthalmichthys nobilis,* and *Hypophthalmichthys molitrix*) were introduced to Honduras for aquaculture purposes (Daniel Mayer personal communication) in the early 1980s and the presence of the Asian fish tapeworm in Honduras may be related to these introductions. However, taking into account the propensity of the Asian fish tapeworm to parasitize species in different families and even orders [8, 31, 32, 35], it is also plausible that the parasite was brought to Honduras through other fish taxa. Additional work is necessary to understand the biogeography and populations genetics of *B. acheilognathi* both in Honduras and elsewhere within its introduced range.

The current distributional area of *B*. *acheilognathi* in Central America includes only two reported localities: one in Panama [9] and one in Honduras (this work). This may be a result of lower sampling intensity in the region, and a more intense and widespread sampling regime may reveal more localities and hosts of *B. acheilognathi*. However, sampling by Aguirre-Macedo et al. [1], and Sandlund et al. [33] failed to record the species from Nicaragua and from Costa Rica. Also, our own sampling failed to record the tapeworm from Guatemala, El Salvador, and two other locations in Honduras. While this suggests that this disjunct pattern of distribution could be real, it seems premature to speculate about the distribution of the Asian fish tapeworm in Central America, because the helminth fauna of the region is poorly studied [30].

This paper provides the first report of *B*. *acheilognathi* in Honduras. The host in this case is *P*. *portillorum*, a new host record, which is a member of a genus faced with a variety of conservation challenges, now complicated by the presence of this pathogenic cestode. In addition, this report documents the disjunct geographical distribution of this invasive parasite in Central America. This paper is also the first report of freshwater fish helminth parasites from Honduras.
